# 4-Amino­benzoic acid–1,2-bis­(4-pyrid­yl)ethane (2/1)

**DOI:** 10.1107/S1600536810020337

**Published:** 2010-06-05

**Authors:** Fwu Ming Shen, Shie Fu Lush

**Affiliations:** a Department of Biotechnology, Yuanpei University, HsinChu, Taiwan 30015; b Department of Medical Laboratory Science Biotechnology, Yuanpei University, HsinChu, Taiwan 30015

## Abstract

In the title compound, C_12_H_12_N_2_·2C_7_H_7_NO_2_, the 4-amino­benzoic acid mol­ecules are linked by O—H⋯N hydrogen bonds to 1,2-bis­(4-pyrid­yl)ethane, forming linear hydrogen bonded chains parallel to [2
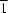
1]. The structure exhibits a hydrogen-bonding network involving COOH⋯N(pyrid­yl)  and amine and carb­oxy­lic N—H⋯ O inter­actions. In addition, π–π stacking inter­actions [centroid–centroid distance = 3.8622 (14) Å] are also present.

## Related literature

For linear hydrogen bonding associations involving 4-amino­benzoic acid, see: Smith *et al.* (1997 ▶). For related structures, see: Smith *et al.* (2000 ▶, 2005 ▶); Lynch & McClenaghan (2001 ▶). For hydrogen-bond motifs, see: Etter *et al.* (1990 ▶). 
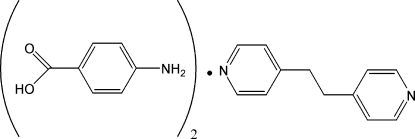


## Experimental

### 

#### Crystal data

C_12_H_12_N_2_·2C_7_H_7_NO_2_
*M* *_r_* = 458.51
Monoclinic, 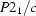

*a* = 7.3556 (10) Å
*b* = 23.230 (3) Å
*c* = 7.9373 (11) Å
β = 115.579 (2)°
*V* = 1223.3 (3) Å^3^
*Z* = 2
Mo *K*α radiation
μ = 0.09 mm^−1^
*T* = 297 K
0.76 × 0.34 × 0.22 mm

#### Data collection

Bruker SMART CCD area-detector diffractometer
Absorption correction: multi-scan (*SMART*; Bruker, 2000 ▶) *T*_min_ = 0.674, *T*_max_ = 1.000
6871 measured reflections
2406 independent reflections
1530 reflections with *I* > 2σ(*I*)
*R*_int_ = 0.032

#### Refinement

*R*[*F* ^2^ > 2σ(*F* ^2^)] = 0.051
*wR*(*F* ^2^) = 0.165
*S* = 1.03
2406 reflections
162 parameters
H atoms treated by a mixture of independent and constrained refinement
Δρ_max_ = 0.21 e Å^−3^
Δρ_min_ = −0.23 e Å^−3^

### 

Data collection: *SMART* (Bruker, 2000 ▶); cell refinement: *SAINT* (Bruker, 1999 ▶); data reduction: *SAINT*; program(s) used to solve structure: *SHELXL97* (Sheldrick, 2008 ▶); program(s) used to refine structure: *SHELXS97* (Sheldrick, 2008 ▶); molecular graphics: *PLATON* (Spek, 2009 ▶); software used to prepare material for publication: *PLATON*.

## Supplementary Material

Crystal structure: contains datablocks global, I. DOI: 10.1107/S1600536810020337/pb2029sup1.cif

Structure factors: contains datablocks I. DOI: 10.1107/S1600536810020337/pb2029Isup2.hkl

Additional supplementary materials:  crystallographic information; 3D view; checkCIF report

## Figures and Tables

**Table 1:** Hydrogen-bond geometry (Å, °)

*D*—H⋯*A*	*D*—H	H⋯*A*	*D*⋯*A*	*D*—H⋯*A*
N1—H1*A*⋯O1^i^	0.90 (3)	2.14 (3)	3.030 (3)	171 (3)
N1—H1*B*⋯O1^ii^	0.93 (3)	2.16 (3)	3.081 (3)	172 (2)
O2—H2*A*⋯N2^iii^	0.90	1.72	2.613 (2)	173

Symmetry codes: (i) 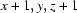; (ii) 
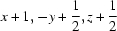
; (iii) 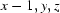.

**Fig. 1.**
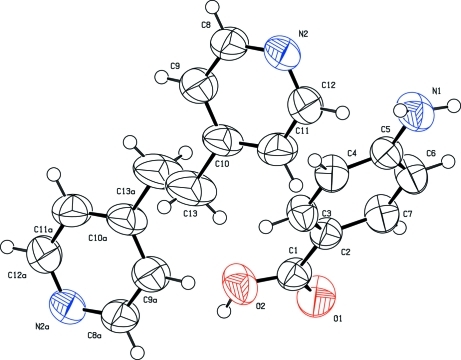
View of the title compound with the atom numbering scheme. Displacement ellipsoids are drawn at the 50% probability level.

**Fig. 2.**
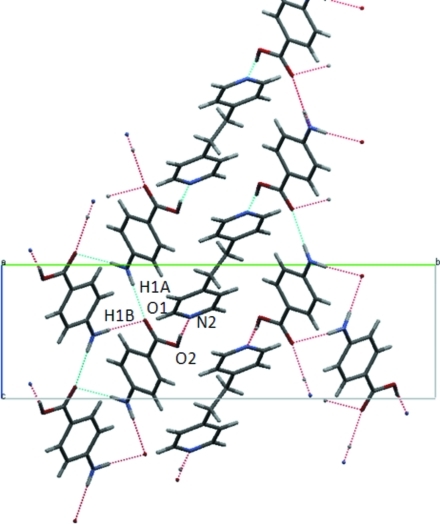
The molecular packing for the title compound, viewed along the a axis. Hydrogen bonds are shown as dashed lines.

## supplementary crystallographic information

### Comment

4-Aminobenzoic acid is a useful ligand for structure extension through both the carboxylic acid and amine functional groups, forming linear hydrogen bonding associations (Smith *et al.*, 2005). Other related reports with 4-aminobenzoic acid and Lewis base such as 4-(4-nitrobenzyl)pyridine (Smith, 1997), 4-aminobenzonitrile (Smith *et al.*, 2000) and 2-amino-4-(4-pyridyl)pyrimidine (Lynch & McClenaghan, 2001).

We present here the crystal structure analysis of the 2:1 4-aminobenzoic acid and 1,2-bis(4-pyridyl)ethane adduct (Fig 1). The structure of the title compound comprises two 4-aminobenzoic acid molecules and one 1,2-bis(4-pyridyl)ethane molecule, with no proton transfer. In the structure, the molecules associate 4-aminobenzoic acid and 1,2-bis(4-pyridyl)ethane via carboxylic and pyridine group O—H···N [ O···N 2.613 (2) Å ] D_2_^2 ^12( Etter *et al.*, 1990), forming linear hydrogen bonding parallel to [ 2 1 1], further connect into a three dimensional network via amine and carboxylic N—H··· O [N···O 3.030 (3) and 3.081 (3) Å ], respectively.

The title compound's supramolecular structure can be readily analyzed in terms of pyridyl atom N2 acts as hydrogen-bond donor to carboxyl atom O2. Similarly, O1 acts as hydrogen-bond donor to amino group N1, respectively (Table 1 and Fig. 2). Furthermore, p -p ring stacking interaction is between neighboring heteraromatic ring in the structure. The distance between Cg1 (N2/C8—C12)···Cg1^i^ is 3.8622 (14) Å[ symmetry code: (i) = 2-X,1-Y,1-Z].

### Experimental

The 4-aminobenzoic acid (0.137 mg, 1.0 mmol) and 1,2-bis(4-pyridyl)ethane (184 mg, 1.0 mmol) were dissolved in 20 ml 50% methanol-water, the solution was refluxed for 30 min. The filtered solution was transferred to a 25 ml tube after one week at room temperature, and colorless transparent crystals formed (yield 60.48%).

### Refinement

N and O-bound H atoms were located in a difference Fourier map and were refined isotropically. Other H atoms were positioned geometrically with C—H=0.93 (aromatic) and 0.97 Å(methylene), and refined using a riding model with Uiso(H)=1.2Ueq(C).

### Crystal data

**Table:**

C_12_H_12_N_2_·2C_7_H_7_NO_2_	*F*(000) = 484
*M**_r_* = 458.51	*D*_x_ = 1.239 Mg m^−^^3^
Monoclinic, *P*2_1_/*c*	Mo *K*α radiation, λ = 0.71073 Å
Hall symbol: -P 2ybc	Cell parameters from 2347 reflections
*a* = 7.3556 (10) Å	θ = 3.0–23.7°
*b* = 23.230 (3) Å	µ = 0.09 mm^−^^1^
*c* = 7.9373 (11) Å	*T* = 297 K
β = 115.579 (2)°	Parallelepiped, colorless
*V* = 1223.3 (3) Å^3^	0.76 × 0.34 × 0.22 mm
*Z* = 2	

### Data collection

**Table:**

Bruker SMART CCD area-detector diffractometer	2406 independent reflections
Radiation source: fine-focus sealed tube	1530 reflections with *I* > 2σ(*I*)
graphite	*R*_int_ = 0.032
phi and ω scans	θ_max_ = 26.1°, θ_min_ = 1.8°
Absorption correction: multi-scan (*SMART*; Bruker, 2000)	*h* = −9→5
*T*_min_ = 0.674, *T*_max_ = 1.000	*k* = −27→28
6871 measured reflections	*l* = −9→9

### Refinement

**Table:**

Refinement on *F*^2^	Primary atom site location: structure-invariant direct methods
Least-squares matrix: full	Secondary atom site location: difference Fourier map
*R*[*F*^2^ > 2σ(*F*^2^)] = 0.051	Hydrogen site location: inferred from neighbouring sites
*wR*(*F*^2^) = 0.165	H atoms treated by a mixture of independent and constrained refinement
*S* = 1.03	*w* = 1/[σ^2^(*F*_o_^2^) + (0.0959*P*)^2^ + 0.0431*P*] where *P* = (*F*_o_^2^ + 2*F*_c_^2^)/3
2406 reflections	(Δ/σ)_max_ < 0.001
162 parameters	Δρ_max_ = 0.21 e Å^−^^3^
0 restraints	Δρ_min_ = −0.23 e Å^−^^3^
0 constraints	

### Special details

**Table:**

Geometry. Bond distances, angles etc. have been calculated using the rounded fractional coordinates. All su's are estimated from the variances of the (full) variance-covariance matrix. The cell esds are taken into account in the estimation of distances, angles and torsion angles
Refinement. Refinement on *F*^2^ for ALL reflections except those flagged by the user for potential systematic errors. Weighted *R*-factors wR and all goodnesses of fit *S* are based on *F*^2^, conventional *R*-factors *R* are based on *F*, with *F* set to zero for negative *F*^2^. The observed criterion of *F*^2^ > σ(*F*^2^) is used only for calculating -*R*-factor-obs etc. and is not relevant to the choice of reflections for refinement. *R*-factors based on *F*^2^ are statistically about twice as large as those based on *F*, and *R*-factors based on ALL data will be even larger.

### Fractional atomic coordinates and isotropic or equivalent isotropic displacement parameters (Å^2^)

**Table:**

	*x*	*y*	*z*	*U*_iso_*/*U*_eq_	
N2	1.1453 (2)	0.43421 (8)	0.3904 (2)	0.0738 (7)	
C8	1.0950 (3)	0.48682 (10)	0.3233 (3)	0.0783 (8)	
C9	0.8987 (3)	0.50396 (11)	0.2231 (4)	0.0864 (9)	
C10	0.7437 (3)	0.46541 (11)	0.1882 (3)	0.0739 (8)	
C11	0.7978 (3)	0.41122 (10)	0.2571 (3)	0.0806 (8)	
C12	0.9970 (3)	0.39735 (10)	0.3566 (3)	0.0826 (9)	
C13	0.5251 (3)	0.48182 (15)	0.0801 (4)	0.1066 (13)	
O1	0.4674 (2)	0.33090 (7)	0.4157 (2)	0.0841 (5)	
O2	0.51671 (19)	0.40480 (7)	0.6066 (2)	0.0837 (6)	
N1	1.3716 (3)	0.28863 (10)	1.0252 (3)	0.0824 (8)	
C1	0.5750 (3)	0.35702 (8)	0.5572 (3)	0.0624 (6)	
C2	0.7798 (3)	0.33843 (8)	0.6832 (2)	0.0560 (6)	
C3	0.8914 (3)	0.36554 (8)	0.8520 (3)	0.0621 (6)	
C4	1.0858 (3)	0.34892 (8)	0.9655 (3)	0.0649 (6)	
C5	1.1749 (3)	0.30406 (8)	0.9143 (3)	0.0601 (6)	
C6	1.0617 (3)	0.27578 (9)	0.7479 (3)	0.0722 (7)	
C7	0.8687 (3)	0.29233 (9)	0.6355 (3)	0.0690 (7)	
H8A	1.19710	0.51350	0.34500	0.0940*	
H9A	0.87020	0.54150	0.17880	0.1040*	
H11A	0.69910	0.38350	0.23650	0.0970*	
H12A	1.02950	0.36010	0.40280	0.0990*	
H13A	0.44700	0.44670	0.03900	0.1280*	
H13B	0.48190	0.50100	0.16510	0.1280*	
H1A	1.413 (4)	0.3021 (12)	1.142 (4)	0.108 (9)*	
H1B	1.409 (3)	0.2543 (12)	0.988 (3)	0.095 (8)*	
H2A	0.38730	0.41220	0.52780	0.1600*	
H3A	0.83380	0.39560	0.88940	0.0750*	
H4A	1.15800	0.36800	1.07780	0.0780*	
H6A	1.11810	0.24500	0.71210	0.0870*	
H7A	0.79550	0.27240	0.52510	0.0830*	

### Atomic displacement parameters (Å^2^)

**Table:**

	*U*^11^	*U*^22^	*U*^33^	*U*^12^	*U*^13^	*U*^23^
N2	0.0497 (10)	0.0777 (12)	0.0824 (12)	0.0081 (8)	0.0176 (9)	0.0039 (9)
C8	0.0490 (11)	0.0770 (14)	0.0935 (16)	−0.0026 (10)	0.0163 (11)	0.0066 (12)
C9	0.0549 (13)	0.0798 (15)	0.1084 (18)	0.0100 (10)	0.0201 (12)	0.0279 (13)
C10	0.0445 (10)	0.0941 (16)	0.0755 (13)	0.0038 (10)	0.0189 (9)	0.0124 (11)
C11	0.0565 (13)	0.0847 (15)	0.0937 (15)	−0.0058 (10)	0.0260 (12)	0.0087 (12)
C12	0.0670 (14)	0.0707 (13)	0.1014 (17)	0.0099 (11)	0.0281 (13)	0.0117 (12)
C13	0.0498 (13)	0.146 (3)	0.110 (2)	0.0138 (13)	0.0212 (13)	0.0438 (17)
O1	0.0607 (8)	0.0805 (10)	0.0741 (9)	−0.0025 (7)	−0.0058 (7)	−0.0085 (8)
O2	0.0553 (8)	0.0796 (10)	0.0888 (11)	0.0151 (7)	0.0052 (7)	−0.0106 (8)
N1	0.0556 (11)	0.0881 (14)	0.0825 (14)	0.0129 (10)	0.0100 (10)	0.0009 (11)
C1	0.0496 (10)	0.0616 (11)	0.0620 (11)	−0.0040 (8)	0.0110 (9)	0.0041 (9)
C2	0.0480 (10)	0.0548 (10)	0.0557 (10)	−0.0022 (8)	0.0134 (8)	0.0004 (8)
C3	0.0574 (11)	0.0591 (11)	0.0592 (11)	0.0088 (8)	0.0152 (9)	−0.0038 (9)
C4	0.0570 (11)	0.0638 (11)	0.0546 (10)	0.0021 (9)	0.0060 (9)	−0.0047 (9)
C5	0.0483 (10)	0.0596 (11)	0.0627 (11)	0.0042 (8)	0.0147 (9)	0.0075 (9)
C6	0.0662 (12)	0.0703 (12)	0.0705 (12)	0.0141 (10)	0.0206 (10)	−0.0085 (10)
C7	0.0628 (12)	0.0690 (12)	0.0593 (11)	0.0029 (9)	0.0114 (9)	−0.0099 (9)

### Geometric parameters (Å, °)

**Table:**

O1—C1	1.220 (3)	C11—H11A	0.9300
O2—C1	1.310 (3)	C12—H12A	0.9300
O2—H2A	0.9000	C13—H13B	0.9700
N2—C12	1.320 (3)	C13—H13A	0.9700
N2—C8	1.321 (3)	C1—C2	1.468 (3)
N1—C5	1.377 (3)	C2—C7	1.390 (3)
N1—H1A	0.90 (3)	C2—C3	1.384 (3)
N1—H1B	0.93 (3)	C3—C4	1.376 (3)
C8—C9	1.373 (4)	C4—C5	1.382 (3)
C9—C10	1.381 (4)	C5—C6	1.386 (3)
C10—C13	1.509 (4)	C6—C7	1.366 (3)
C10—C11	1.362 (3)	C3—H3A	0.9300
C11—C12	1.369 (3)	C4—H4A	0.9300
C13—C13^i^	1.436 (4)	C6—H6A	0.9300
C8—H8A	0.9300	C7—H7A	0.9300
C9—H9A	0.9300		
			
O1···N1^ii^	3.081 (3)	C12···H4A^xi^	3.0000
O1···N1^iii^	3.030 (3)	C13···H9A^i^	2.7900
O2···N2^iv^	2.613 (2)	H1A···O1^vii^	2.14 (3)
O1···H1A^iii^	2.14 (3)	H1A···H4A	2.3000
O1···H1B^ii^	2.16 (3)	H1B···H6A	2.3200
O1···H7A	2.5700	H1B···O1^viii^	2.16 (3)
O1···H11A	2.9200	H1B···C1^viii^	2.81 (3)
O1···H4A^iii^	2.8000	H2A···C8^iv^	2.6900
O2···H3A	2.4500	H2A···C12^iv^	2.6200
O2···H8A^v^	2.7400	H2A···N2^iv^	1.7200
O2···H13B^vi^	2.8400	H3A···O2	2.4500
N1···O1^vii^	3.030 (3)	H3A···C11^xii^	3.0600
N1···O1^viii^	3.081 (3)	H4A···H1A	2.3000
N2···O2^ix^	2.613 (2)	H4A···C12^xii^	3.0000
N2···C1^ix^	3.368 (3)	H4A···O1^vii^	2.8000
N1···H6A^x^	2.9500	H6A···H1B	2.3200
N2···H2A^ix^	1.7200	H6A···N1^xiii^	2.9500
C1···N2^iv^	3.368 (3)	H6A···C4^xiii^	2.8700
C3···C9^v^	3.567 (3)	H6A···C5^xiii^	2.8100
C8···C9^v^	3.587 (4)	H7A···O1	2.5700
C9···C8^v^	3.587 (4)	H8A···O2^v^	2.7400
C9···C3^v^	3.567 (3)	H9A···C4^v^	2.8700
C1···H1B^ii^	2.81 (3)	H9A···C13^i^	2.7900
C3···H9A^v^	2.8600	H9A···C3^v^	2.8600
C4···H9A^v^	2.8700	H9A···H13A^i^	2.2400
C4···H6A^x^	2.8700	H11A···O1	2.9200
C5···H6A^x^	2.8100	H11A···H13A	2.3500
C7···H12A	3.0300	H12A···C7	3.0300
C8···H2A^ix^	2.6900	H13A···H11A	2.3500
C9···H13A^i^	2.7500	H13A···C9^i^	2.7500
C11···H3A^xi^	3.0600	H13A···H9A^i^	2.2400
C12···H2A^ix^	2.6200	H13B···O2^vi^	2.8400
			
C1—O2—H2A	110.00	C13^i^—C13—H13A	108.00
C8—N2—C12	117.08 (19)	C13^i^—C13—H13B	108.00
C5—N1—H1A	111 (2)	O2—C1—C2	114.69 (17)
C5—N1—H1B	113.1 (14)	O1—C1—O2	122.1 (2)
H1A—N1—H1B	128 (2)	O1—C1—C2	123.22 (19)
N2—C8—C9	122.9 (2)	C1—C2—C7	120.41 (16)
C8—C9—C10	119.9 (2)	C3—C2—C7	117.52 (19)
C9—C10—C11	116.5 (2)	C1—C2—C3	122.07 (19)
C11—C10—C13	121.1 (2)	C2—C3—C4	121.4 (2)
C9—C10—C13	122.4 (2)	C3—C4—C5	120.6 (2)
C10—C11—C12	120.2 (2)	N1—C5—C4	120.6 (2)
N2—C12—C11	123.3 (2)	C4—C5—C6	118.1 (2)
C10—C13—C13^i^	117.1 (2)	N1—C5—C6	121.3 (2)
C9—C8—H8A	118.00	C5—C6—C7	121.1 (2)
N2—C8—H8A	119.00	C2—C7—C6	121.17 (19)
C8—C9—H9A	120.00	C2—C3—H3A	119.00
C10—C9—H9A	120.00	C4—C3—H3A	119.00
C10—C11—H11A	120.00	C3—C4—H4A	120.00
C12—C11—H11A	120.00	C5—C4—H4A	120.00
N2—C12—H12A	118.00	C5—C6—H6A	119.00
C11—C12—H12A	118.00	C7—C6—H6A	119.00
C10—C13—H13B	108.00	C2—C7—H7A	119.00
H13A—C13—H13B	107.00	C6—C7—H7A	119.00
C10—C13—H13A	108.00		
			
C12—N2—C8—C9	0.3 (3)	O2—C1—C2—C3	7.6 (3)
C8—N2—C12—C11	0.0 (3)	O2—C1—C2—C7	−172.38 (19)
N2—C8—C9—C10	−0.2 (4)	C1—C2—C3—C4	−177.7 (2)
C8—C9—C10—C11	−0.3 (4)	C7—C2—C3—C4	2.3 (3)
C8—C9—C10—C13	179.6 (2)	C1—C2—C7—C6	177.6 (2)
C9—C10—C11—C12	0.6 (3)	C3—C2—C7—C6	−2.3 (3)
C13—C10—C11—C12	−179.3 (2)	C2—C3—C4—C5	−0.5 (3)
C9—C10—C13—C13^i^	40.3 (4)	C3—C4—C5—N1	178.0 (2)
C11—C10—C13—C13^i^	−139.9 (3)	C3—C4—C5—C6	−1.2 (3)
C10—C11—C12—N2	−0.4 (3)	N1—C5—C6—C7	−178.0 (2)
C10—C13—C13^i^—C10^i^	−180.0 (2)	C4—C5—C6—C7	1.2 (3)
O1—C1—C2—C3	−173.5 (2)	C5—C6—C7—C2	0.6 (3)
O1—C1—C2—C7	6.5 (3)		

Symmetry codes: (i) −*x*+1, −*y*+1, −*z*; (ii) *x*−1, −*y*+1/2, *z*−1/2; (iii) *x*−1, *y*, *z*−1; (iv) *x*−1, *y*, *z*; (v) −*x*+2, −*y*+1, −*z*+1; (vi) −*x*+1, −*y*+1, −*z*+1; (vii) *x*+1, *y*, *z*+1; (viii) *x*+1, −*y*+1/2, *z*+1/2; (ix) *x*+1, *y*, *z*; (x) *x*, −*y*+1/2, *z*+1/2; (xi) *x*, *y*, *z*−1; (xii) *x*, *y*, *z*+1; (xiii) *x*, −*y*+1/2, *z*−1/2.

### Hydrogen-bond geometry (Å, °)

**Table:**

*D*—H···*A*	*D*—H	H···*A*	*D*···*A*	*D*—H···*A*
N1—H1A···O1^vii^	0.90 (3)	2.14 (3)	3.030 (3)	171 (3)
N1—H1B···O1^viii^	0.93 (3)	2.16 (3)	3.081 (3)	172 (2)
O2—H2A···N2^iv^	0.90	1.72	2.613 (2)	173

Symmetry codes: (vii) *x*+1, *y*, *z*+1; (viii) *x*+1, −*y*+1/2, *z*+1/2; (iv) *x*−1, *y*, *z*.
